# Sequence and Structural Analysis of AA9 and AA10 LPMOs: An Insight into the Basis of Substrate Specificity and Regioselectivity

**DOI:** 10.3390/ijms20184594

**Published:** 2019-09-17

**Authors:** Xiaoli Zhou, Xiaohua Qi, Hongxia Huang, Honghui Zhu

**Affiliations:** State Key Laboratory of Applied Microbiology Southern China, Guangdong Provincial Key Laboratory of Microbial Culture Collection and Application, Guangdong Microbial Culture Collection Center (GDMCC), Guangdong Institute of Microbiology, Guangdong Academy of Sciences, Guangzhou 510070, China; zhouxl@gdim.cn (X.Z.); qixiaohua7@163.com (X.Q.); huanghx6470@163.com (H.H.)

**Keywords:** lytic polysaccharide monooxygenases, LPMOs, substrate specificity, regioselectivity

## Abstract

Lytic polysaccharide monooxygenases (LPMOs) are key enzymes in both the natural carbon cycle and the biorefinery industry. Understanding the molecular basis of LPMOs acting on polysaccharide substrates is helpful for improving industrial cellulase cocktails. Here we analyzed the sequences, structures, and substrate binding modes of LPMOs to uncover the factors that influence substrate specificity and regioselectivity. Our results showed that the different compositions of a motif located on L2 affect the electrostatic potentials of substrate binding surfaces, which in turn affect substrate specificities of AA10 LPMOs. A conserved Asn at a distance of 7 Å from the active center Cu might, together with the conserved Ser immediately before the second catalytic His, determine the localization of LPMOs on substrate, and thus contribute to C4-oxidizing regioselectivity. The findings in this work provide an insight into the molecular basis of substrate specificity and regioselectivity of LPMOs.

## 1. Introduction

Lytic polysaccharide monooxygenases (LPMOs) are a group of copper-dependent enzymes that catalyze the oxidatively cleavage of glycoside bonds in polysaccharides, such as cellulose and chitin [1,2]. The ability of these enzymes to boost the degradation of crystalline polysaccharides represents a promising resource for development of industrial enzyme cocktails for biomass processing [3,4]. LPMOs are distributed in seven families (AA9, AA10, AA11, AA13, AA14, AA15 and AA16) in the auxiliary activities classes of CAZy (Carbohydrate-Active enZYmes) database (www.cazy.org) based on sequence similarity [5,6]. Members of AA9 are cellulose-active LPMOs from fungi, with C1- and/or C4-oxidizing activity [7]. AA10 family is more complex in terms of species origin and substrate specificity. Members of AA10 are derived from archaea, bacteria, fungi or viruses, some of which act on chitin and others on cellulose. In AA10 family, all of the chitin-active LPMOs are C1-oxidizing, while some cellulose-active LPMOs are C1-oxidizing and others are both C1- and C4-oxidizing. There is no cellulose-active LPMOs oxidizing C4 carbon only found in AA10 family [8]. Members of AA11 [9], AA13 [10] and AA14 [11] are mainly from fungi with chitin-, starch- and xylan- activity, respectively. AA15 [12] are from virus and eukaryote (including animals), with cellulose- or chitin-activity. AA16 are fungal LPMOs with cellulose-activity [6].

Similar to other carbohydrate active enzymes, LPMOs are also modular enzymes [13]. A certain percentage of LPMOs are associated with various CBMs (carbohydrate binding module) or GHs (glycoside hydrolase) domains. Some LPMOs appended CBMs have been described to affect substrate interaction, and influence the enzyme activity and stability [14,15]. However, for different LPMOs, CBM domains seemly have different effects on substrate binding, since they have been reported to enhance, weaken or not affect the enzyme performance of LPMOs [16,17]. The catalytic domain of LPMOs is an immunoglobulin-like β-sandwich of seven to nine β-strands. The active site contains a copper ion coordinated by two conserved histidines, one of which is the N terminal histidine, in an arrangement called “histidine brace”. Multiple extended loops (L2, L3, LS and LC) connect the core β strands, shaping the substrate binding surface [13,18]. The sequences and topologies of these loops are highly diverse, which is thought to be related to the different substrate specificities. It is speculated that the solvent-exposed aromatic residues on the substrate binding surface are responsible for substrate binding [8,19,20].

The structures of LsAA9A (a C1/C4-oxidizing LPMO) complex with cellulose polysaccharides and the recent molecular dynamics simulation studies of the binding modes of LPMOs with crystalline polysaccharide substrates have provided us with key information about the structure–function relationship of LPMOs [21,22,23]. However, the mechanism of the C1- or C4-oxidation regioselectivity is not yet fully understood. The active site geometry of a LPMO on a specific substrate is considered to be the cause of oxidation regioselectivity [2,24]. Some substructure features have been proposed to influence substrate binding and thus affect the regioselectivity, such as the L2 loop, which is an important part of the substrate binding surface [25]; the serine/threonine immediately before the second histidine ligand to the copper active site, which may influence the orientation of the copper center on substrate [26]; and the axial tyrosine ligand (Y166 in NcLPMO9C), which might involve in the electron transfer [18,26]. In addition, the posttranslational glycosylation may also play a role in substrate binding [27]. So far, a large number of highly diverse LPMOs sequences have been identified, but only a few enzyme activities and structures have been characterized, which hinders our understanding of the structural and functional relationship of LPMOs.

In this study, to investigate the determinants of substrate specificity and oxidation regioselectivity, we analyzed the sequences and structures of all characterized LPMOs in AA9 and AA10 families (too few LPMOs are characterized in other families). We found that the composition of a motif on L2 plays an important role in substrate specificity. A conserved Asn residue at 7Å from catalytic center Cu is involved in the localization of the enzyme on substrate, and then contributes to C4-oxidation, while the appended domains are related to substrate specificity but not regioselectivity.

## 2. Results

### 2.1. A Motif on L2 Loop Affects the Substrate Specificity of AA10 LPMOs

**Figure 1:**
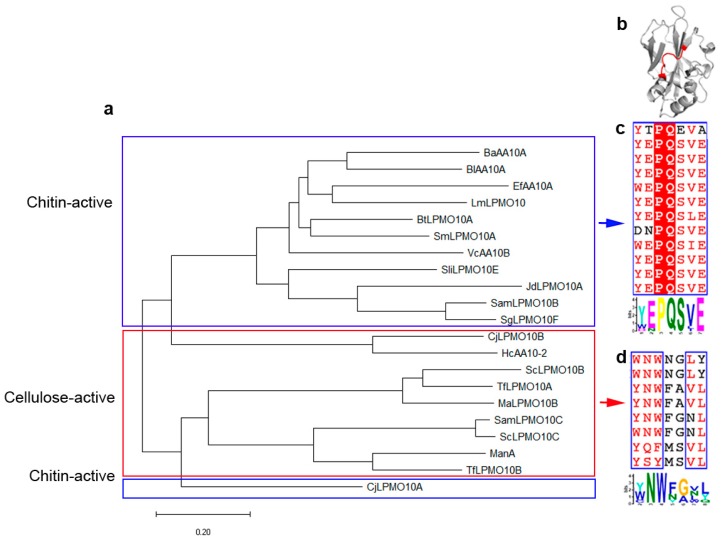
Sequence analysis of characterized lytic polysaccharide monooxygenases (LPMOs) in AA10 family. (**a**) phylogenetic tree for AA10 LPMOs created by MEGA-X. The members in the blue and red boxes are chitin-active and cellulose-active LPMOs, respectively. (**b**) The location of the two motifs (red) on L2 loop, taking the structure of CjLPMO10A as an example (Protein Data Bank (PDB) entry: 5fjq [29]). (**c**,**d**) Multi-sequence alignments and the motif logos of chitin-active and cellulose-active LPMOs, respectively.

Unlike other families, AA10 LPMOs have diverse substrate specificities, some of which act on chitin, while others act on cellulose. To study the factors affecting substrate specificity, we first analyzed the sequences of catalytic domain of characterized AA10 LPMOs (a total of 20 sequences from bacteria in CAZy database until 24 July 2019). These sequences are classified into two groups in the phylogenetic tree (Figure 1a). The first group are mainly chitin-active with two exceptions: HcAA10 and CjLPMO10B are characterized as cellulose-active. The second group are mainly cellulose-active, except that CjLPMO10A is chitin-active. By multiple sequence alignment and MEME (Multiple Em for Motif Elicitation) search [28], we found two different motifs at the corresponding positions on L2 loop in chitin-active and cellulose-active LPMOs (Figure 1b). In chitin-active LPMOs, more than 70% residues of the motif (Y(W)EPQSVE) are polar amino acids, especially the two negatively charged glutamic acids (Figure 1c). In contrast, in cellulose-active LPMOs, the motif (Y(W)NWFGVL) contains more than 70% hydrophobic amino acids (Figure 1d).

**Figure 2:**
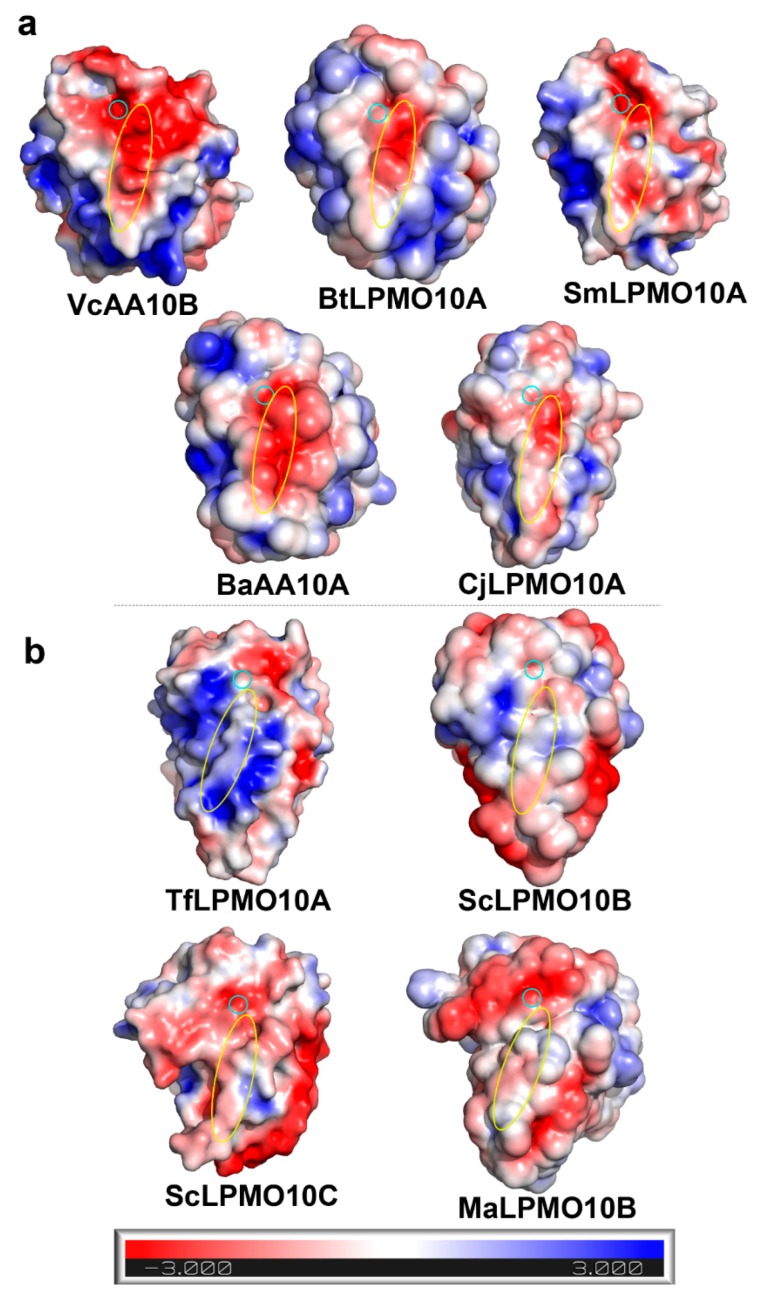
Electrostatic potentials of substrate binding surfaces. (**a**,**b**) Chitin-active and cellulose-active AA10 LPMOs, respectively. The electrostatic potential ranges from −3 to +3. Red indicates negatively charged, blue indicates positively charged, white indicates uncharged. Blue circle indicates the site of catalytic center Cu. Yellow circle indicates the stretch of the motif on L2. The PDB entries for the structures used here are 2xwx [30], 5wsz, 2bem [31], 2yow [32], 4gbo [17], 4oy6 [33], 4oy7 [33], 5opf [34]. The electrostatic potentials were calculated by PDB2PQR [35] and the figures were produced by PyMOL.

Since these two motifs are located on L2, an important component of the substrate binding surface, we wondered at the effect of their different amino acid compositions on substrate specificity. We calculated the surface electrostatic potentials of different LPMOs at pH 6, which is commonly used to detect LPMOs activity. As shown in Figure 2, the substrate binding surfaces of chitin-active LPMOs are negatively charged (Figure 2a), which is favorable for enzymes binding to chitin surface with positive charge. While the substrate binding surfaces of cellulose-active LPMOs are uncharged or positively charged (Figure 2b), which may be more suitable for the binding of uncharged cellulose surface.

### 2.2. A Conserved Asparagine at 7Å of Catalytic Cu Contributes to C4-Oxidation

**Figure 3:**
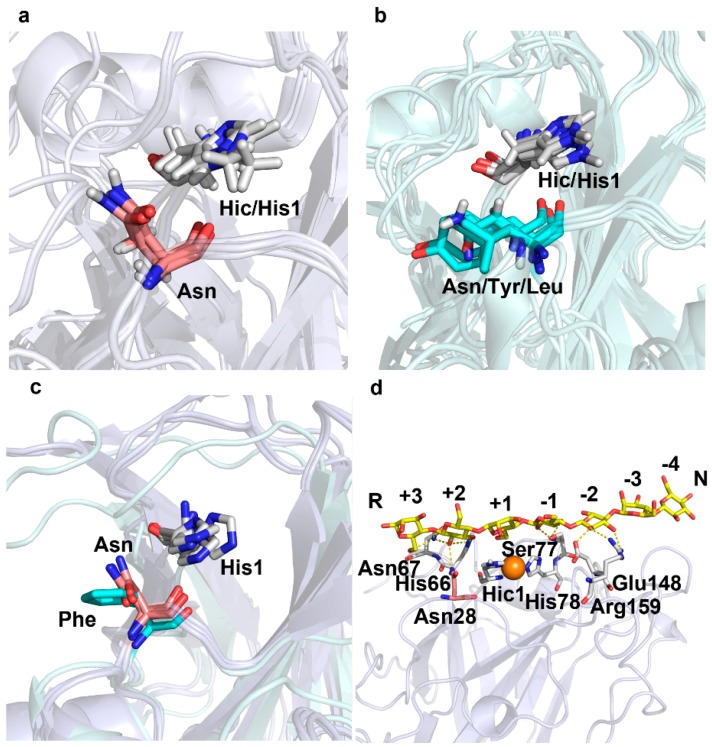
Structural comparison between C4- or C1/C4-oxidizing LPMOs and C1-oxidizing LPMOs in AA9 and AA10. (**a**) C4- or C1/C4-oxidizing AA9 LPMOs. PDB entries for protein structures used here are: 5nkw [22], 4d7u [36], 4eir [19], 4eis [19], and 5foh [34]. (**b**) C1-oxidizing AA9 LPMOs. PDB entries for protein structures used here are: 2yet [37], 4b5q [38], 4qi8 [39], 5nns [24], and 5ufv [40]. (**c**) Cellulose-active AA10 LPMOs. PDB entries for protein structures used here are: 4gbo [17], 4oy6 [33], and 4oy7 [33]. (**d**) Structure of LsAA9A complexed with cellulose polysaccharide substrate (PDB entry: 5nkw). The conserved Asn residues are shown in pink sticks, the residues on the corresponding sites of C1-oxidizing LPMOs are shown in cyan sticks, the catalytic His and other residues involved in substrate binding are shown in white sticks, the substrate is shown in yellow sticks. The H-bonds between enzyme and substrate are shown in yellow dashed lines.

To investigate the factors influencing oxidation regioselectivity, we compared the structures of LPMOs with different regioselectivities to find their characteristics. Interestingly, we found a conserved Asn residue (Asn 28 of LsAA9A) on L2 of C4-oxidizing or C1/C4-oxidizing LPMOs in AA9 family (Figure 3a). These Asn residues are about 5 Å away from the catalytic His1 and about 7 Å from the catalytic center Cu. The side chains of these Asn are perpendicular to the substrate binding surfaces, pointing to substrate. However, in C1-oxidizing LPMOs, the corresponding sites are not conserved, and hydrophobic Tyr and Leu appear in addition to Asn (Figure 3b). Moreover, the side chains of these residues are parallel to the substrate binding surfaces, even if the residue on this site is Asn. We also compared the structures of cellulose-active LPMOs in AA10 family (Figure 3c). Both LPMOs with C1/C4-oxidizing activity (TfLPMO10A and ScLPMO10B) in AA10 contain the conserved Asn at the corresponding sites, with the same side chain orientations as AA9 family. While in the C1-oxidizing LPMO (ScLPMO10C), the corresponding site is a Phe with the phenyl ring parallel to the substrate binding surface. From the crystal structure of LsAA9A complexed with cellulose polysaccharide substrate (Protein Data Bank (PDB) entry: 5nkw), we can find that Asn28 participates in substrate binding by hydrogen bonding with O2 atom of +2 glucose subunit (Figure 3d).

**Figure 4:**
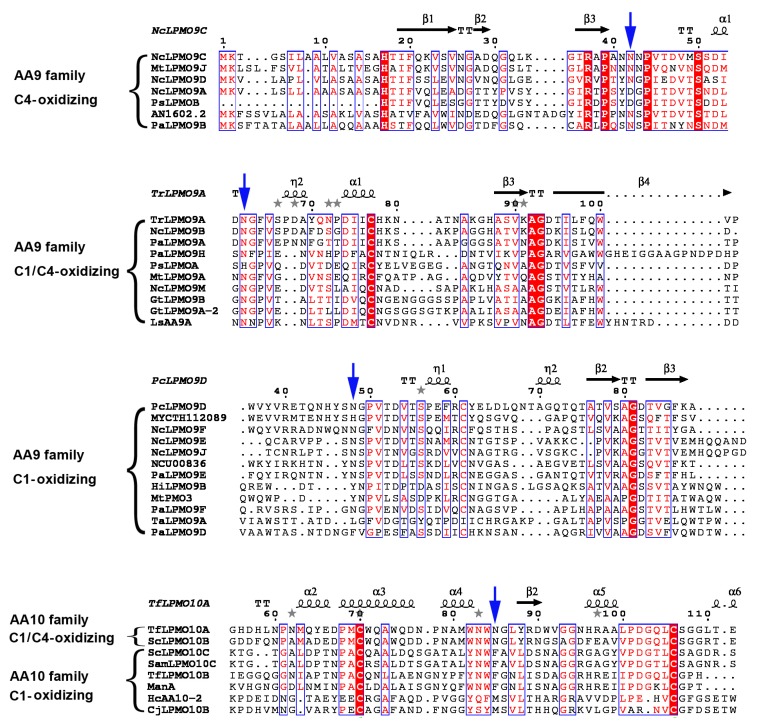
Multiple sequence alignments of characterized AA9 and AA10 LPMOs. The highly conserved amino acid residues, which are conserved in >70%, are highlighted in red letters. The residues conserved in 100% are highlighted with a red background. The blue arrows indicate the conserved Asn in C4-oxidizing LPMOs or the corresponding site in C1-oxidizing LPMOs. The figure was produced using ESPript3.0 [41].

In order to identify whether this phenomenon is common in other LPMOs without crystal structures, we performed multi-sequence alignment of all the characterized cellulose-active LPMOs in AA9 and AA10 families (Figure 4). For C4- and C1/C4-oxidizing LPMOs, this site is conserved (Asn/Asp) with the only exception of PsLPMOA, the corresponding site of which is His. For AA9 C1-oxidizing LPMOs, amino acids such as His, Leu, Tyr and Phe appear at this site besides Asn. For AA10 C1-oxidizing LPMOs, residues on this site are hydrophobic Phe or Met.

**Figure 5:**
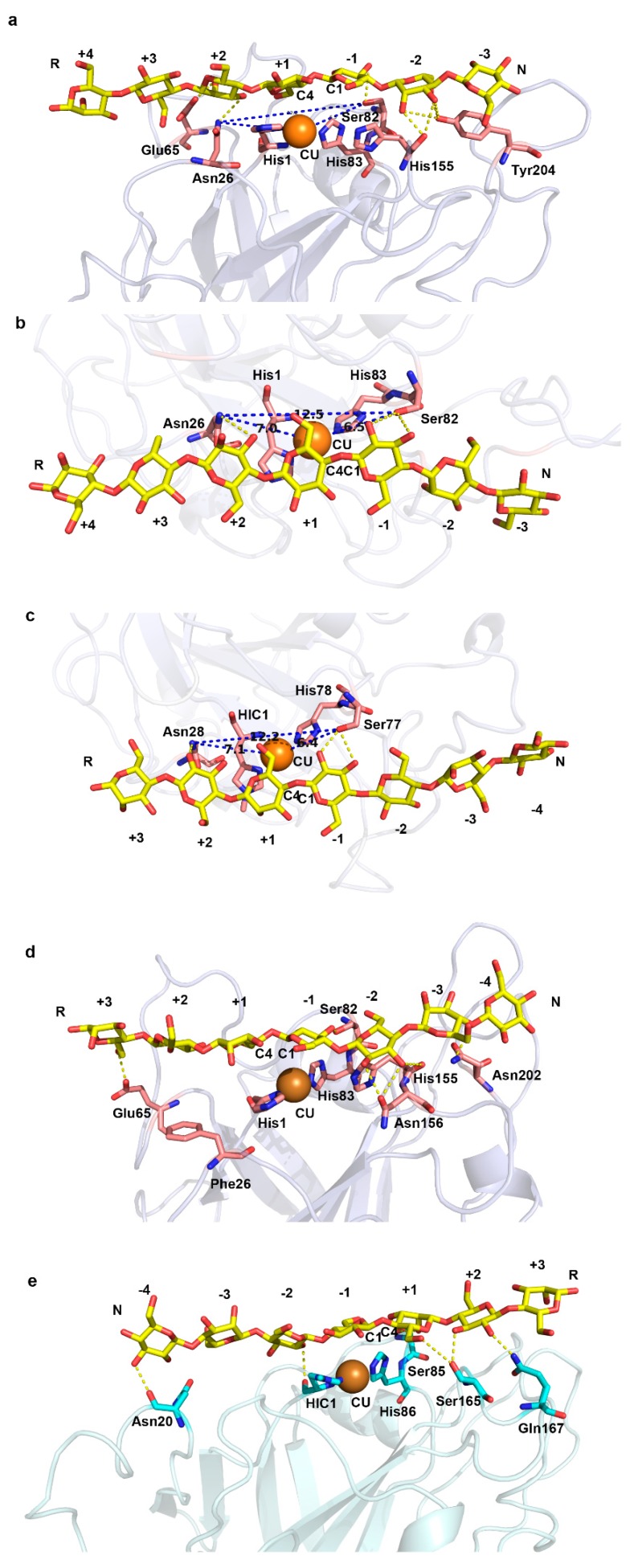
Substrate binding modes of C1-oxidizing and C4-oxidizing LPMOs with substrate G7 predicted by molecular docking. (**a**) NcLPMO9C–G7 complex. (**b**,**c**) are top view of NcLPMO9C–G7 and LsAA9A–G7 complex, respectively. (**d**) NcLPMO9C_N26F–G7 complex. (**e**) TaLPMO9A–G7 complex. G7 is shown in yellow sticks. Cu is shown in orange spheres. Catalytic His and the residues involved polar interactions with substrate are shown in sticks. The polar interactions between enzyme and substrate are shown in yellow dashed lines. The distances between residues are labeled and shown in blue dashed lines. The orientations of G7 are labeled with R (reduced end) and N (nonreduced end).

The above findings inspired us to investigate the role of this conserved Asn near the catalytic center on substrate binding patterns. We extracted the polysaccharide molecule (G7) in the crystal structure of LsAA9A (PDB entry: 5nkw), and docked it into C4-oxidizing LPMO (NcLPMO9C, PDB entry: 4d7u) and C1-oxidizing LPMO (TaLPMO9A, PDB entry: 2yet), respectively (Figure 5 and Table 1). The binding mode of NcLPMO9C–G7 complex (Figure 5a) is similar to that of LsAA9A (Figure 3d). The Asn26 forms a pair of H-bond with G7, between its ND2 atom and the O2 atom of sugar unit in the putative +2 subsite. Meanwhile, the OG atom of Ser82 forms a pair of H-bond with O2 and O3 of sugar unit in the putative −1 subsite, respectively. Asn26, Cu and Ser82 are arranged in a line parallel to the substrate polysaccharide chain (Figure 5b). The distance between Asn26 and Ser82 is 12.5 Å, and that between these two residues and Cu is 7 Å and 6.5 Å, respectively. Similarly, the corresponding distances in LsAA9A–G7 complex structure are 12.2 Å, 7.1 Å and 6.4 Å, respectively (Figure 5c). We hypothesize that this arrangement and the hydrogen bonds formed by the two residues (Asn26 and Ser82 of NcLPMO9C; Asn28 and Ser77 of LsAA9A) with substrates place Cu in an appropriate position for the oxidation of C4 carbon (Table 1).

**Figure 6:**
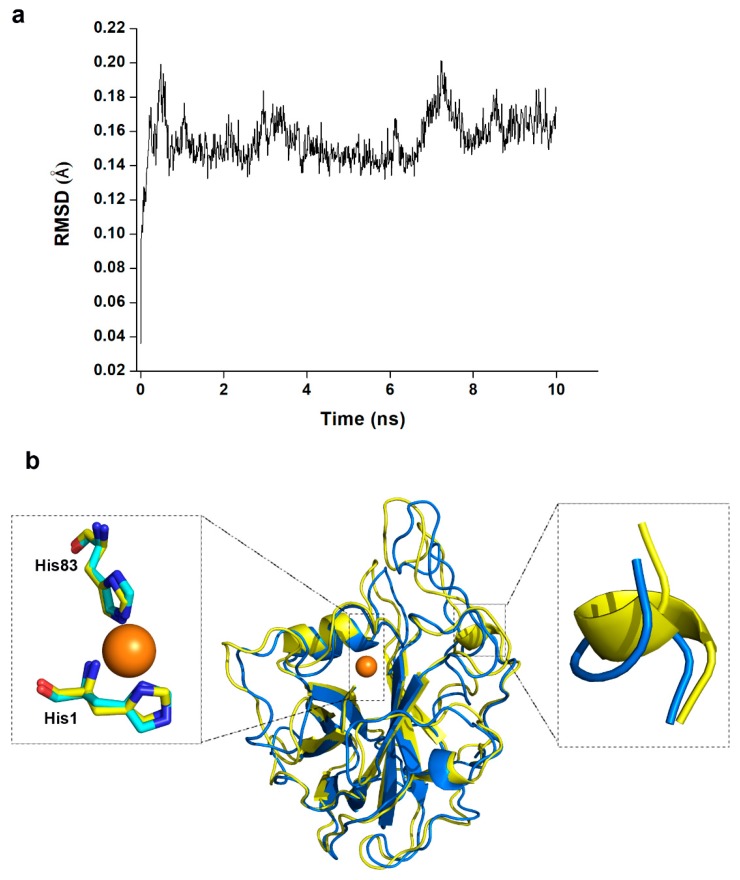
Structure model of NcLPMO9C_N26F. (**a**) Average backbone RMSD of 10 ns molecular dynamic simulation of the structure model. (**b**) Superposition of the average structure of NcLPMO9C_N26F (yellow) with the crystal structure of NcLPMO9C (PDB entry: 4d7u) (blue). The catalytic centers and the loops of residues 196–201 are circled and shown in the enlarged diagrams.

To test our hypothesis, we mutated the Asn26 of NcLPMO9C into Phe (an amino acid common in C1-oxidizing LPMOs) in silico. After homology modelling, the model structure was performed with a 10 ns molecular dynamic simulation (Figure 6). The trajectories of the model reached equilibrium after 2 ns, and the RMSD stabilized with time (Figure 6a). We calculated the average structure between 4 ns and 6 ns as a structure model of the mutant for subsequent studies. The overall structure and catalytic center of the mutant did not change much compared to the wild type (Figure 6b). The most distinct region was the loop of residues 196–201, which folded into a short helix in the mutant. This loop region was not directly involved in interactions with substrate in the molecular docking results of NcLPMO9C (Figure 5a).

Then we docked G7 into the structure model of the mutant. The complex model is shown in Figure 5d, and the polar interactions between the mutant enzyme and substrate are listed in Table 1. The orientation of the polysaccharide chain was not changed, but the position relative to Cu shifted (Table 1). The shift of the substrate binding position increased the distance between catalytic Cu and C4 carbon from 4.0 Å to 5.1 Å, while decreasing the distance between Cu and C1 carbon from 5.0 to 4.4 Å. The changes of these distances may change the regioselectivity or reduce the C4-oxidizing activity of the enzyme. This result indirectly confirms the effect of the arrangement of Asn26 and Ser82 on substrate binding mode and C4-oxidizing regioselectivity.

In TaLPMO9A–G7 complex model, the orientation of polysaccharide chain is opposite to that of LsAA9A and NcLPMO9C (Figure 5e). The OG atom of Ser85 (corresponding to Ser77 of LsAA9A and Ser82 of NcLPMO9C) forms an H-bond with O2 and O3 of the sugar unit in the putative +1 subsite, respectively. The Leu41 (corresponding to Asn28 of LsAA9A and Asn26 of NcLPMO9C) interacts with the sugar units in –2 and –3 subsites through van der Waals forces. Other polar interactions between TaLPMO9A and substrate are listed in Table 1. The distances between Cu and C1 or C4 of the scissile glycosidic bond are 4.2 Å and 5.6 Å, respectively, which is more favorable for oxidation of C1 carbon.

### 2.3. Appended Modules Are Related to Substrate Specificity but Not Regioselectivity

**Figure 7:**
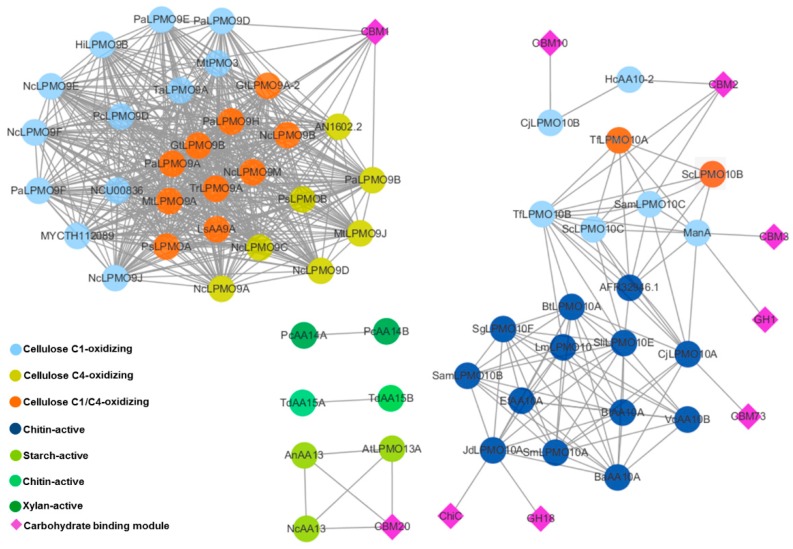
Sequence and domain similarity networks of characterized LPMOs. Circles represent enzymes, and colors represent different substrate specificities or regioselectivities. Diamonds represent appended modules. Edges represent BLAST similarity with a bit score greater than 200 (e-value > e^−50^). The figure was produced by Cytoscape [42].

One characteristic of carbohydrate-active enzymes is modularity. Like other families, a large proportion of LPMOs have additional domains. To investigate the influence of module composition on the substrate specificity and oxidation regioselectivity, we analyzed the sequence and domain similarity of all the characterized LPMOs in CAZy database. From the sequence and domain similarity network in Figure 7 we can find that there is no connections between families. Regardless of the regioselectivity, the AA9 LPMOs are either single catalytic domain or appended with CBM1 domains, which are found almost exclusively in fungi and are characterized by cellulose-binding. Whereas the appended domains of AA10 are more diverse: The cellulose-active LPMOs such as CjLPMO10B, HcAA10, and ManA are appended with cellulose-binding modules, namely the CBM10, CBM2, CBM3 and GH1, while the chitin-active LPMOs such as CjLPMO10A and JdLPMO10A are appended with chitin-binding modules, namely the CBM73, ChiC and GH18. The three characterized AA13 LPMOs are all associated with starch-binding CBM20. The currently characterized AA14 and AA15 LPMOs are all single catalytic domain. AA11 and AA16 LPMOs are not shown in this network since there is only one enzyme characterized in each family.

## 3. Discussion

In this study we analyzed the sequences, structures, and substrate binding modes of characterized LPMOs in AA9 and AA10 families, to investigate the molecular basis of substrate specificity and oxidation regioselectivity.

The catalytic domains of LPMOs have similar β-sandwich core structures, but the loops outside the core structures are highly diverse [13]. Studies have shown that these diverse loops constitute substrate binding surfaces, and that some key residues on substrate binding surfaces play an important role in substrate binding [43]. By sequence alignment, we found two different motifs on L2 loop of AA10 LPMOs with different substrate specificities. The motifs stretch along substrate binding surfaces and are close to the catalytic centers. In chitin-active LPMOs, the compositions of the motif are mainly polar amino acids, and under common catalytic conditions (pH 6), the electrostatic potentials of the motif stretch region are negative. Whereas in cellulose-active LPMOs, the motifs are mainly composed of hydrophobic amino acids, and the electrostatic potentials of this region are positive or uncharged. We speculate that the different amino acid compositions of these different motifs, and thus the different electrostatic potentials, make LPMOs suitable for binding to positively charged chitin substrates or uncharged cellulose substrates, respectively.

Through sequence and structural alignment, we found a conserved Asn on L2 of C4- or C1/C4-oxidizing LPMOs. This Asn and the conserved Ser immediately before the second catalytic His are located on opposite sides of the catalytic Cu, at a distance of 7Å and 6.5 Å, respectively. These three are arranged in a line parallel to the orientation of the substrate polysaccharide chain. Asn and Ser form H-bonds with the +2 and –1 sugar subunits of the substrate, respectively. This arrangement, like a vernier caliper, positions the enzyme on the surface of the substrate and places the catalytic Cu in a position suitable for C4 oxidation.

Mutation of Asn to Phe did not change the substrate orientation, but shifted its binding position relative to catalytic center, which in turn affected the distances between Cu and the C4 or C1 carbon of the scissile glycosidic bond. Changes in these distances may alter the regioselectivity of the enzyme or may only reduce the activity of C4-oxidation. Consistent with this result, Forsberg et al. reported that mutating Asn at the corresponding position of MaLPMO10B to Phe (N85F) reduced the proportion of C4-oxidation product [34]. These results indicate that Asn28 is a key factor, but not the only factor of C4-oxidizing activity.

We calculated the pKa values of residues of NcLPMO9C and the mutant N26F at pH6.0 (Table 2). The pKa values of His1 and His83 were slightly decreased from 3.66 to 3.51 and 1.47 to 1.39, respectively. Compared with the wild type, the most obvious change in pKa value was Glu65, from 4.66 to 5.01. This may be due to changes in the environment of the Glu65 side chain, caused by the conformational change of the flexible loop where the Glu65 is located during the molecular dynamic simulation. Since there was almost no change in pKa of other residues on the substrate binding surface, we speculate that the N26F mutation may not alter substrate specificity of the enzyme. N85F mutation did not completely abolish the C4-oxidizing activity of MaLPMO10B in ref 35, also indicating that the N85F mutation had no or little effect on substrate specificity.

From the sequence and domain similarity networks, for AA9 and AA10 families, the substrate specificities of the appended modules are consistent with that of the whole LPMOs. However, the appended modules are not related to the regioselectivities of LPMOs. Since there are too few characterized LPMOs of other families available for similarity network analysis, it is not currently possible to infer the roles of the additional modules in those families. The substrate specificities and oxidation regioselectivities of LPMOs are complicated, and more research is needed to understand its molecular basis.

## 4. Conclusions

In summary, the present study suggests that LPMOs form different substrate binding surface characteristics during evolution, including electrostatic potentials and the arrangement of key amino acids, which determine their substrates preference and localization on substrate surface. The findings in this study are helpful for further understanding the molecular basis of substrate specificity and regioselectivity of LPMOs.

## 5. Materials and Methods

### 5.1. Multiple Sequence Alignment and Phylogenetic Tree Construction

Protein sequences of characterized AA10 LPMOs were obtained from the National Center for Biotechnology Information (NCBI) protein database (https://www.ncbi.nlm.nih.gov/), and aligned by ClustalW (https://www.ebi.ac.uk/Tools/msa/clustalw2) [44]. Aligned sequences were then trimmed to retain only the catalytic domains. Phylogenetic trees were generated using MEGA-X [45] by neighbor-joining method. The figures of multiple sequence alignment were produced using ESPript3.0 (http://espript.ibcp.fr/ESPript/ESPript/).

### 5.2. Motif Identification

The program MEME version 5.0.5 (http://meme-suite.org/tools/meme) was used to identify amino acid sequence motifs. The sequences of cellulose-active LPMOs or chitin-active LPMOs were input into the web server. MEME was run, allowing zero or one site per sequence, and the motif width was restricted to 6–10 amino acids. Common sequence motifs were selected, matching with *p* value <0.0001.

### 5.3. Electrostatic Potential and pKa Value Calculation

Electrostatic potentials of the LPMOs surfaces, without any ligand, were calculated using the program APBS [46]. The PQR file and the pKa values were calculated via the PDB2PQR server (http://nbcr-222.ucsd.edu/pdb2pqr_2.1.1/) specifying the pH at 6, which is the commonly used pH for detecting activity.

### 5.4. Protein Structure Analysis and Molecular Docking

Protein structures were obtained from the Protein Data Bank (http://www.rcsb.org/), and superimposed by using PyMOL (The PyMOL Molecular Graphics System, Version 1.8 Schrödinger, LLC, De Lano Scientific, San Carlos, CA, USA).

The cellulose substrate G7 was extracted from the complex structure of LsAA9A (PDB entry: 5nkw). The PDB entries for structures of NcLPMO9C and TaLPMO9A used for docking were 4d7u and 2yet, respectively. AutoDock 4.2.6 (The Scripps Research Institute, La Jolla, CA, USA) [47] was used for molecular docking study. The AutoDock Tools 1.5.6 was used to prepare the protein and ligands for docking procedure. All solvent molecules, water molecules, and the cocrystallized ligands were removed from the structures. Kollman charges and polar hydrogens were added. AutoGrid was used to generate the grid maps. Each grid was centered at the substrate binding surface of the LPMOs. The grid dimensions were 126 points in each dimension separated by 0.375 Å. The files were generated as PDBQT format. For ligand G7, random starting positions and orientations were used, and no torsion was allowed. The genetic algorithm was used with 2,500,000 energy evaluations and a population of 150 individuals, and 100 runs were carried out.

### 5.5. Homology Modelling and Molecular Dynamic Simulation

The homology model of mutant NcLPMO9C_N26F was created by SWISS-MODEL (https://swissmodel.expasy.org/) [48], using the structure of NcLPMO9C (PDB entry: 4d7u) as template.

The molecular dynamic simulation was performed by Gromacs 2018 [49]. In order to simulate a physiological environment, physiological saline was added with an explicit periodic boundary model. The GROMOS96 43a1 force-field was used. The protein atoms were energy-minimized by applying 1000 steps of steepest descent. The temperature of the system was slowly driven from 50 to 300 K. The production was performed for 10 ns at a constant temperature of 300 K and a constant pressure, and the results were saved at a frequency of 0.002 ns. The coordinate of His1, His83 and Cu were restrained. The average structure was calculated by Gromacs.

### 5.6. Sequence Similarity Network

Sequence similarity network was produced according to the methods reported in ref 8. Sequences of all characterized LPMOs in CAZy database were compared against each other using BLAST (https://blast.ncbi.nlm.nih.gov/Blast.cgi). Domain structures of each protein were annotated with CAZy database using dbCAN server (http://bcb.unl.edu/dbCAN2/) [50]. Then, the data was used to build a similarity network using Cytoscape 3.7.1 (Free Software Foundation Inc., Boston, MA, USA) [42].

## Figures and Tables

**Table 1:** Interaction Parameters of the Complexes Predicted by Molecular Docking.

Complex	Polar Contacts	Distance 1 (Å) ^a^	Distance 2 (Å) ^b^
NcLPMO9C–G7	BMA322:O3-Asn26:ND2 BMA322:O2-Glu65:OE2 BGC324:O3-Ser82:OG BMA325:O2-His155:O BMA325:O3-His155:O BMA325:O2-Tyr204:OH BMA325:O3- Tyr204:OH BMA325:O6- Tyr204:OH	5.0	4.0
NcLPMO9C_N26F–G7	BMA320:O6- Glu65:OE2 BGC323:O3-Ser82:OG BGC324:O2-Asn156:OD1 BGC324:O3-Asn156:OD1 BGC324:O3-His155:O BMA325:O4-Asn202:ND2	4.4	5.1
TaLPMO9A–G7	BGC326:O3-Asn20:O BGC324:O2-HIC:O BMA322:O2-Ser85:OG BMA322:O3-Ser85:OG BMA322:O6-Ser165:OG BGC321:O3- Ser165:OG BGC321:O2-Gln167:NE2	4.2	5.6

^a^ Distance between Cu and C1 carbon of sugar unit in –1 subsite; ^b^ Distance between Cu and C4 carbon of sugar unit in +1 subsite.

**Table 2:** pKa Values of Residues of NcLPMO9C and Mutant N26F at pH6.

Residue	pKa		Residue	pKa	
	WT	N26F		WT	N26F
Asp 13	2.59	2.59	Tyr 90	13.49	13.49
Asp 31	3.38	3.38	Tyr 133	10.47	10.47
Asp 36	4.35	4.35	Tyr 145	12.01	11.98
Asp 46	2.69	2.69	Tyr 166	11.99	12.02
Asp 74	3.19	3.19	Tyr 192	18.6	18.59
Asp 76	3.86	3.86	Tyr 204	10.64	10.64
Asp 95	3.23	3.23	Tyr 217	14.13	14.13
Asp 123	2.61	2.61	Lys 6	11.06	11.06
Asp 124	4.49	4.49	Lys 18	10.43	10.43
Asp 135	3.96	3.96	Lys 45	9.58	9.58
Asp 196	4.15	4.15	Lys 57	10.55	10.55
Asp 211	3.09	3.09	Lys 84	10.77	10.77
Glu 65	4.66	5.01	Lys 93	9.55	9.55
Glu 112	4.59	4.58	Lys 106	10.38	10.38
Glu 150	4.37	4.37	Lys 109	9.54	9.54
His 1	3.66	3.51	Lys 119	10.55	10.55
His 60	3.44	3.44	Lys 209	10.5	10.5
His 64	6.85	6.83	Lys 215	10.2	10.2
His 83	1.47	1.39	Arg 21	10.95	10.95
His 155	4.31	4.28	Arg 148	14.86	14.86
